# The Number Needed to Immunize (NNI) to Assess the Benefit of a Prophylaxis Intervention with Monoclonal Antibodies Against RSV

**DOI:** 10.3390/vaccines13080791

**Published:** 2025-07-25

**Authors:** Sara Boccalini, Veronica Gironi, Primo Buscemi, Paolo Bonanni, Barbara Muzii, Salvatore Parisi, Marta Borchiellini, Angela Bechini

**Affiliations:** 1 Dipartimento di Scienze della Salute, Università degli Studi di Firenze, 50134 Firenze, Italy; paolo.bonanni@unifi.it (P.B.); angela.bechini@unifi.it (A.B.); 2 Scuola di Specializzazione in Igiene e Medicina Preventiva, Università degli Studi di Firenze, 50134 Firenze, Italy; veronica.gironi@unifi.it (V.G.); primo.buscemi@unifi.it (P.B.); 3 Sanofi, Viale L. Bodio, 37/b, 20158 Milan, Italy; barbara.muzii@sanofi.com (B.M.); salvatore.parisi@sanofi.com (S.P.); marta.borchiellini@sanofi.com (M.B.)

**Keywords:** prevention, respiratory syncytial virus, number needed to vaccinate, children, respiratory disease, passive immunoprophylaxis, nirsevimab

## Abstract

**Introduction:** Respiratory Syncytial Virus (RSV) is the leading cause of lower respiratory tract infections in infants and children, as well as hospitalizations for respiratory infections in the pediatric population, representing a significant public health concern. Nirsevimab, a long-acting anti-RSV monoclonal antibody, has recently been approved by the European Medicines Agency (EMA). The aim of this study is to assess the utility of certain parameters, such as the Number Needed to Immunize (NNI), in supporting decision-makers regarding the introduction of nirsevimab as a universal prophylactic measure. **Methods**: A literature review was conducted to identify the definition and application of the NNI in the context of infectious disease prevention. The following online databases were consulted: Scopus, MEDLINE, Google Scholar, Web of Science, and Cochrane Library. The search was restricted to English-language texts published between 1 January 2000 and 30 January 2025. **Results**: The NNI represents the number of individuals who need to be immunized to prevent clinical outcomes such as medical visits and hospitalizations caused by infectious diseases. Six studies were identified that utilized this parameter to outline the benefits of immunization and describe the advantages of using monoclonal antibodies for RSV disease. Finelli and colleagues report that to prevent one RSV-related hospitalization, 37–85 infants aged 0–5 months and 107–280 infants aged 6–11 months would need to be immunized with long-acting anti-RSV antibodies. A recent study by Mallah et al. on the efficacy of nirsevimab estimates that the NNI required to prevent one RSV-related hospitalization is 25 infants. Studies by Francisco and O’Leary report NNI values of 82 and 128 infants, respectively, to prevent one RSV-related hospitalization with nirsevimab. Mallah et al. describe NNI as a metric useful to quantify the immunization effort needed to prevent a single RSV hospitalization. A recent Italian study reports that 35 infants need to be immunized to prevent one hospitalization due to RSV-LRTI and 3 infants need to be immunized to prevent one primary care visit due to RSV-LRTI. The studies indicate that the NNI for anti-RSV monoclonal antibodies is lower than the corresponding Number Needed to Vaccinate (NNV) for vaccines already included in national immunization programs. The main limitations of using this parameter include the absence of a shared threshold for interpreting results and the lack of consideration for the indirect effects of immunization on the population. **Conclusions:** The NNI is an easily understandable tool that can be used to convey the value of an immunization intervention to a variety of stakeholders, thereby supporting public health decision-making processes when considered in association with the uptake of the preventative strategy. At the current status, the estimated NNI of monoclonal antibodies against RSV results favourable and confirms the use in the first year of life for the prevention of RSV disease.

## 1. Introduction

Respiratory syncytial virus (RSV) is the most common cause of lower respiratory tract infections (LRTIs) in infants and young children, including bronchiolitis and pneumonia [1]. RSV infections contribute to approximately 60% to 80% of cases of bronchiolitis in infants and up to 40% of cases of pneumonia in children [2,3]. Furthermore, children with RSV-induced bronchiolitis are at increased risk of developing medium- and long-term respiratory complications, such as asthma and recurrent wheezing [4,5]. Although premature infants and children with chronic diseases are at higher risk for severe RSV-related illness, most hospitalizations due to RSV occur in healthy, full-term infants [6].

RSV represents a significant burden for health-care systems. Globally, a recent review estimated 33 million RSV-associated LRTI episodes, 3.6 million hospitalizations, and 120,000 deaths caused by RSV in children under five years annually [7]. The Italian surveillance network RespiVirNet for the 2023–2024 season reported that RSV-positive samples were 16% of all positive samples for respiratory viruses under surveillance in the entire population. The majority of cases were observed among patients aged 0–2 years [8]. The reason for the dramatic epidemiological impact of RSV can be attributed to its high contagiousness, as it has a transmissibility index (R0) of 4.5, higher than the influenza virus (R0 = 1.2) and second only to the measles virus (R0 = 18) [7].

A key strategy for RSV prevention involves monoclonal antibodies, such as palivizumab and nirsevimab. Palivizumab is a monoclonal antibody approved for immunoprophylaxis in a limited high-risk infant population, leaving the majority of infants vulnerable to RSV [9,10,11]. Most recently, nirsevimab, a long-lasting monoclonal antibody, received approval in the European Union for the prevention of lower respiratory tract diseases caused by RSV in newborns and infants during their initial RSV season [12]. Nirsevimab is able to protect children for at least 5 months with a single administration, reducing hospitalization for RSV respiratory infections by 77% and the risk of Intensive Care Unit (ICU) admission by 86% [13].

Countries that already implemented a universal immunization campaign with nirsevimab demonstrated a relevant public health impact in terms of reduction of the burden of RSV. In particular, in the United States of America, the Centre for Disease Control estimated an effectiveness of 93% against RSV hospitalization [14]. In Spain [15,16,17,18,19] and Chile [20,21], due to the high coverage achieved around 90%, this prevention strategy caused a dramatic public health impact with about 90% reduction of RSV hospitalization compared to previous seasons. Although there are no data regarding mortality rate reduction, WHO estimated that the implementation of a universal prevention strategy with nirsevimab will reduce it dramatically, ultimately saving many infant lives globally [22]. Moreover, as of today, >6 million doses of nirsevimab have been distributed and administered worldwide, and data from this real-world experience confirmed the good safety profile [21,23,24,25,26,27,28,29].

Currently in Italy, nirsevimab has not been included in the National Immunization Plan [30]. However, its inclusion was recommended by the Board of the Calendar for Life and the Italian Society of Neonatology as a universal immunoprophylaxis for RSV infections in all infants at their first RSV season [31]. Since the RSV season 2024/2025, all regions of Italy have implemented a preventive strategy with nirsevimab to protect all infants, with a specific fund provided by the Ministry of Health [32].

The aim of this study is to identify whether parameters, like the Number Needed to Immunize (NNI), can be useful in assessing and supporting the decision to introduce and implement the use of nirsevimab as a preventive measure for all newborns and children in the National Immunization Plan in Italy. Particularly, this review addresses a specific knowledge gap that extends beyond merely summarizing existing literature. This manuscript focuses on exploring and evaluating the potential of NNI as a comprehensible and reliable metric to support policymakers in their decisions, rather than just compiling existing NNI values. While other reviews might present NNI values, this study’s explicit aim is to assess the utility of certain parameters in supporting decision-makers regarding the introduction of nirsevimab as a universal prophylactic measure in a specific policy-oriented context. It moves to an evaluation of the metric’s practical value in policy-making. In addition, this review does not just present NNI values but also thoroughly performs a critical analysis of NNI’s strengths and limitations as a tool. By identifying and discussing the different methodologies used in included studies, even when descriptions are scarce, the manuscript implicitly contributes to a clearer understanding of this aspect, which is a recognized gap in the NNI literature.

## 2. Materials and Methods

### 2.1. Search Strategy

We reviewed the literature to better understand the definition of NNI and its application in helping policymakers in the assessments for the introduction of public health measures. Particularly, we performed a comprehensive narrative literature review to assess the utilization extent, variation in application, and interpretation of the NNI metric. This review encompassed only English-language articles published between 1 January 2000 and 30 January 2025. The search terms employed were “number needed to immunize” and “NNI”. The following online databases were searched: Scopus, MEDLINE, Web of Science, Cochrane Library (CENTRAL, Cochrane Database of Systematic Reviews), and Google Scholar.

Particularly, studies on NNI applied to monoclonal antibodies against RSV disease were researched.

### 2.2. Study Selection

The authors independently conducted an initial screening of the titles and abstracts of all studies identified through the search. Articles that mentioned the terms “NNI” or “number needed to immunize” within the full text were included. Studies not meeting the inclusion criteria were excluded at this stage. Subsequently, the authors performed a detailed evaluation and appraisal of the full texts of the remaining articles. This review imposed no restrictions on study design. A narrative synthesis was undertaken due to the methodological heterogeneity and variability in result presentations. Particularly, the definition and context of application of NNI, calculation of NNI, and the main results of the included studies on NNI applied to monoclonal antibodies against RSV disease were analysed.

## 3. Results

### 3.1. Search Results

Eight articles [19,33,34,35,36,37,38,39] met the inclusion criteria. Among these, 6 studies used this parameter to delineate the benefits of immunization and describe the advantages of monoclonal antibody use in RSV disease [19,35,36,37,38,39]. The remaining two studies address the NNI parameter in relation to other diseases rather than discussing RSV [33,34].

### 3.2. Definition and Context of Application of NNI

NNI is defined as the number of individuals that need to be immunized to prevent one outcome and is a corollary measure for the Number Needed to Vaccinate (NNV), defined as the number of persons needed to be vaccinated in order to prevent one outcome [40].

While vaccination and immunization may be often used interchangeably in general discourse, they have a crucial difference in this context. The intervention associated with NNV is explicitly a vaccine, which provides active prevention. NNI is a broader measure; critically, the intervention for NNI can be the administration of either a vaccine or an antibody, thus encompassing both active and passive prophylaxis strategies. This study focuses particularly on the NNI for nirsevimab. Therefore, in this context, “immunize” primarily refers to the administration of such an antibody. In summary, the key difference is that NNI is a more inclusive term that covers both active prophylaxis (via vaccines) and passive prophylaxis (via monoclonal antibodies), whereas NNV is specifically tied to the administration of vaccines. This allows NNI to be applied to novel prophylactic measures like monoclonal antibodies, broadening its utility beyond traditional vaccines. NNI is a measure that is currently underutilized in the literature. Armstrong et al. used the NNI to prevent one case or death from the disease to comment on the recommendations for meningococcal vaccination [33].

Soderstrom et al., in a study aimed at describing the rate of overwhelming post-splenectomy infections (OPSI) in patients with asplenia, utilized the NNI to summarize the results of their study regarding the efficacy of vaccinations in asplenic individuals [34].

Finelli et al. described NNI as a corollary measure for the NNV, defining the NNI as the number of infants that need to be immunized to prevent one RSV-associated outpatient visit, outpatient lower respiratory infection (LRI) or hospitalization [35].

In their article offering recommendations for the administration of nirsevimab to prevent RSV disease, Francisco et al. highlight the benefits of its use by applying metrics such as the NNI to prevent one hospitalization, as well as to prevent hospitalization associated with severe disease [36].

O’Leary et al. considered the NNI with nirsevimab to prevent specific health outcomes, including outpatient visits, emergency department visits, hospital admissions, and ICU admissions [37].

Mallah et al. (Lancet Infect Dis. 2024) calculated the NNI as the inverse of the number of cases averted per one case, referring to the estimated number of RSV-related LRTI hospitalizations prevented directly by the nirsevimab immunization campaign, to avoid one case of RSV-associated LRTI hospitalization [19].

Mallah et al. (Hum Vaccin Immunother. 2024) described the method used for estimating the NNI in the NIRSE-GAL study, stating that the NNI was estimated based on the absolute risk reduction, incidence rate, or the estimated averted cases, selecting the most appropriate measure depending on the number of events [38]. The study by Mallah et al. employed the NNI metric to evaluate the effectiveness of nirsevimab in preventing various RSV-related hospitalizations.

A recent Italian study evaluating the seasonal epidemiological and economic impact of RSV by comparing the current prophylaxis strategy using palivizumab in eligible infants with a universal immunization approach using nirsevimab also estimated the NNI needed to prevent one RSV-related event per season [39].

Therefore, from the literature review we conducted, it has been revealed that the NNI was employed to quantify the number of individuals required to be immunized to prevent various clinical outcomes, including hospitalization, hospitalization combined with severe disease, admission to intensive care units, disease, or death. In a similar way to the NNV, NNI is used to delineate the benefits of vaccination and to describe the advantages of using monoclonal antibodies (Table 1).

Table 1 delineates key distinctions among three population-based metrics—NNT (Number Needed to Treat), NNV (Number Needed to Vaccinate), and NNI (Number Needed to Immunize)—each serving as a quantifiable measure for assessing the effectiveness of preventive or therapeutic interventions in clinical and public health settings. Specifically, the NNT is defined as the number of patients requiring treatment to avert one adverse outcome, with medication serving as the intervention and recovery as the primary outcome [40]. In contrast, the NNV and NNI apply to healthy individuals as the target population, where vaccination acts as the intervention. However, the NNI extends beyond vaccination to include antibody administration, broadening its applicability to both active and passive prevention strategies. These distinctions allow for tailored application across varied clinical and epidemiological contexts, with NNT focusing on therapeutic recovery and NNV and NNI geared toward preventative outcomes.

### 3.3. Calculating NNI

Few studies have employed the NNI to describe the benefits of immunization strategies, and the methods used in these studies for calculating the NNI are heterogeneous and often poorly described.

Finelli et al. calculated the NNI as the reciprocal of the absolute incidence rate reduction (1/ARR). The ARR was derived by multiplying the incidence rates of RSV-associated events by the immunization efficacy. The 95% CIs for NNI were determined as the reciprocal of the ARR 95% CI. Specifically, the lower limit of NNI was 1 divided by the upper limit of ARR, and the upper limit of NNI was 1 divided by the lower limit of ARR [35].

In the study by Mallah et al. (Hum Vaccin Immunother. 2024), a Poisson regression model with robust variance was used to estimate nirsevimab’s effectiveness in preventing RSV-related hospitalizations. Adjusted incidence rate ratios (IRR) and their 95% CI were calculated, taking into account the enrollment group and sex. The effectiveness of nirsevimab was expressed as (1 − IRR) × 100. Cox proportional hazard models were used to estimate hazard ratios (HR), with effectiveness calculated as (1 − HR) × 100. The NNI was determined based on absolute risk reduction, incidence rate, or averted cases, depending on which measure was most appropriate given the number of events [38].

In Marcellusi et al., while an explicit mathematical formula for NNI (e.g., 1/ARR) is not stated, the estimation within the model, based on efficacy, implies a calculation method consistent with the inverse of the intervention’s impact. For instance, it reports that 3 infants would need to be immunized to prevent one RSV MA-LRTI and 35 infants for one RSV-LRTI hospitalization, given the stated efficacy [39].

In summary, while Finelli et al. [35] provide a clear formula (NNI = 1/ARR), Mallah et al. [38] describe a broader conceptual approach for its estimation, and Marcellusi et al. [39] present NNI values as outputs from a model incorporating efficacy. The other studies we evaluated did not provide detailed descriptions of the methodologies employed to calculate the NNI. 

### 3.4. Summary of the Results of the Studies on NNI Applied to Monoclonal Antibodies Against RSV Disease

Five studies [19,35,36,37,39] have calculated NNI as a parameter to evaluate the efficacy of monoclonal antibodies against RSV disease, as summarized in Table 2. The NNI values vary depending on the specific outcome measured, the age group targeted, and the assumed immunization efficacy, reflecting the differing contexts of the studies.

Finelli et al. in their study elucidated the benefits of an extended half-life monoclonal antibody (EHL-mAb) for the prevention of RSV infection in pediatric populations in the United States [35]. They varied immunization efficacy (IE) from a low of 50% to a high of 90% to account for its undetermined nature at the time of the study, using 70% as a mid-range reference point. For an EHL-mAb with 70% efficacy, 6–18 infants need to be immunized to prevent one RSV-associated outpatient visit in the first year of life. Similarly, an EHL-mAb with 70% efficacy would require immunizing 13–33 infants to prevent one RSV-associated lower respiratory tract infection (LRI) outpatient visit in the first year of life. Regarding RSV-associated hospitalizations, the NNI to prevent one RSV-associated hospitalization for infants in the first year of life through an EHL-mAb of 70% efficacy is 37–280, with approximately three times as many 6–11-month-old infants needing immunization compared to those aged 0–5 months. More details are reported in Table 3.

The analysis of Finelli et al. demonstrated that immunizing infants with an RSV EHL-mAb, even with moderate efficacy, could have a substantial impact. Moreover, the estimated NNI for the RSV EHL-mAb was lower than the NNVs reported for various other vaccines included in the routine childhood immunization program in the United States [35]. In a statement issued by the Spanish Society of Paediatric Infectious Disease (SEIP), Francisco et al. offered recommendations regarding the use of nirsevimab for prevention of RSV disease. The article evaluated the use of the extended half-life monoclonal antibody (EHL-mAb) nirsevimab for preventing RSV infections in children, supporting the routine administration of nirsevimab to all infants, particularly those born during the RSV season or under six months at the season’s onset, to reduce the incidence and the severity of RSV-related hospitalizations. The authors reported the results of a trial conducted during the 2022–2023 RSV season, which included 8058 infants up to 12 months old (4037 received nirsevimab and 4021 received the standard of care). The study found that the NNI was 82 to prevent one hospitalization and 285 to prevent hospitalization combined with severe disease, showing benefits consistent with previous trials. The panel, based on this evidence, particularly recommended administration to infants born during the RSV season or aged less than 6 months at its onset, as well as to high-risk groups [36].

The study of O’Leary et al. provides an update from the Advisory Committee on Immunization Practices (ACIP) regarding the prevention of respiratory syncytial virus (RSV) and other vaccine recommendations. It highlighted the unanimous recommendation for the use of nirsevimab for RSV prevention in infants, including those born during the RSV season or less than 6 months old at its onset. O’Leary et al. highlighted that the NNI of nirsevimab for various health outcomes compared favorably with other routine vaccines: 17 for outpatient visits, 48 for emergency department visits, 128 for hospital admissions, and 581 for ICU admissions. It was also noted that nirsevimab would be cost-effective in the second season due to its significantly lower price compared to palivizumab [37].

The NIRSE-GAL study, conducted in Galicia, Spain, assessed the effectiveness of the monoclonal antibody nirsevimab for preventing RSV-related hospitalizations in infants. The study estimated that a median of 30 infants (IQR 23–30) in the overall cohort, or 16 infants (12–17) in the seasonal cohort, would need to be immunized to prevent one RSV-related LRTI hospitalization. It also demonstrated significant protection against all-cause LRTI and hospitalizations, with no severe adverse events reported. These findings provide robust, real-world evidence to inform policymakers on RSV prevention strategies [19].

In a recent study conducted in Italy, a static decision analytic model was used to assess the health and cost outcomes related to RSV, comparing the use of nirsevimab to the current standard of care (SoC) in preventing medically attended RSV-related lower respiratory tract infections (RSV-MA-LRTIs). The model also assessed the public health impact of nirsevimab by estimating the NNI to prevent a single RSV-related event per season. Assuming a 79.50% efficacy of nirsevimab against RSV MA-LRTI, the findings indicated that immunizing 3 newborns would prevent one RSV MA-LRTI case, 35 would be needed to prevent one RSV-LRTI hospitalization, 241 to prevent one ICU admission due to RSV-LRTIs, 16 to prevent one ER visit due to RSV-LRTIs, 3 to prevent one primary care visit due to RSV-LRTIs, and 24,942 to prevent one inpatient death caused by RSV-LRTIs [39].

These results clearly indicate that the NNI for anti-RSV monoclonal antibodies tends to be lower (meaning greater impact) for less severe outcomes, such as outpatient or primary care visits, and higher for more severe outcomes, such as hospitalizations, ICU admissions, or deaths. The NNI also varies with the age group (e.g., lower NNI for hospitalization in younger infants due to higher incidence in that group) and the efficacy assumed in the modeling. For example, the Italian study by Marcellusi et al. [39], assuming a nirsevimab efficacy of 79.50% against RSV-MA-LRTI, shows the wide range of NNIs from 3 (for primary care visits and RSV-MA-LRTI) to 24,942 (for inpatient death).

## 4. Discussion

The aim of the study was to review the literature to better understand the definition and current use of NNI and to understand whether it can be useful in supporting policymakers to introduce nirsevimab in immunization plans. Four studies have been identified that used this parameter to outline the benefits of immunization and describe the advantages of using monoclonal antibodies in RSV disease (and one study is in progress). Furthermore, from the literature analysis, the limitations and advantages of using NNI were identified, along with comparisons with currently employed immunization strategies.

### 4.1. Limitations of NNV and NNI

Since NNI is a measure similar to NNV and is often calculated in the same manner, the limitations identified for interpreting NNV also apply to NNI. The principal limitation in using these parameters is that NNV does not consider the indirect effects of vaccination, such as the reduction in secondary transmission resulting from fewer infectious individuals and the benefits of herd immunity (Table 4). However, the omission of these indirect effects might be insignificant when dealing with non-transmissible vaccine-preventable diseases, such as tetanus. However, for communicable diseases, vaccination’s impact is highly non-linear, and the indirect effects of vaccination can greatly surpass the direct effects accounted for in NNV calculations [41].

The study of Tuite and Fisman reported that, in a variety of simulated infectious diseases with different epidemiological characteristics, NNV calculations resulted in estimates with biases of up to three orders of magnitude, equivalent to a 1000-fold difference. The extent of the distortion is greater for diseases with low R0, longer time horizons, and in the presence of vaccines with low efficacy [42]. However, this is not our case because it should be considered that the basic reproduction number of RSV is particularly high. On the other hand, Tuite’s study concluded that the use of NNV can underestimate the potential benefits of vaccination programs, especially in terms of the benefit for public health. In addition, even if a high-efficacy preventive tool (vaccine or monoclonal antibodies) is available, the possible public health impact can be assessed considering immunization coverage in the target population.

Another limitation in using NNV and NNI is the absence of defined thresholds for what is a favorable number, which complicates the interpretation and generalizability of this measure and makes it difficult to compare different prevention strategies [43].

Furthermore, the methods used for calculating NNI are often heterogeneous and poorly described across different studies. This methodological variability can make direct comparisons of NNI values from different research or health systems problematic, impacting the generalizability of findings. For all these reasons, NNV and NNI calculations need to be applied with caution and interpreted critically when employed as metrics for assessing the potential community-level impact of vaccination and immunization programs.

### 4.2. Advantages of NNV and NNI

The main advantage of using NNI and NNV is their ability to describe the effort required by immunization programs for generating a significant public health impact in terms of reduction of disease burden, assuming that uptake and immunization/vaccination coverage is high among the target population. As a matter of fact, the programs that require a lower NNV/NNI are more advantageous than those that require a higher one [43]. NNV and NNI can also be used as parameters to demonstrate the cost-effectiveness ratio of a particular immunization program, and based on this information, formulate a policy recommendation [43]. NNI is an easy and intuitive tool that can help policymakers and health authorities in making decisions [43]. Finally, this parameter, together with the achievement of high coverage among the target population, can be useful to understand the effort required to generate a significant public health impact through immunization strategies in terms of reduction of disease burden.

Table 4 summarizes the main strengths and limitations of using NNI and NNV.

### 4.3. Comparison of NNI with NNV of Some Vaccination Strategies

The estimated NNI for nirsevimab was lower compared to NNV for vaccines targeting other pathogens included in the routine childhood immunization schedule. For instance, to prevent one influenza-related hospitalization, 1000–3000 children aged 6–23 months would need to be vaccinated with a vaccine having 50% efficacy, compared to 149–392 infants 6–12 months of age that would need to be immunized with an extended half-life RSV monoclonal antibody (EHL-mAb) with 50% efficacy. Additionally, 12–42 children would need to be vaccinated to prevent one influenza-related outpatient visit, compared to 8–22 infants that would need to be vaccinated with an EHL-mAb [44]. In addition, the NNV to prevent one case of influenza is 43 (16–192) for people aged 65 years and over, and the NNV to prevent one hospitalization is 777 (470–1684), which are higher than the NNI for nirsevimab [40].

Regarding rotavirus vaccination, it is estimated that 200 infants should be vaccinated with the pentavalent rotavirus vaccine with 85% efficacy to prevent a single rotavirus-associated hospitalization. In the case of an RSV EHL-mAb with 70% efficacy, the estimated number of infants that should be vaccinated to prevent one RSV-related hospitalization ranges from 37 to 280 [45].

Similarly, NNV for pneumococcal disease is substantially higher than the NNI for EHL-mAb. A recent study estimated that 671 infants must be vaccinated with the pneumococcal conjugate vaccine to prevent one case of invasive pneumococcal disease, while 448 children under 36 months would need immunization to prevent one case of consolidated community-acquired pneumonia [46]. Additionally, 16,524 persons (13,770–22,533) aged 65 years and older should be vaccinated with the 13-valent pneumococcal conjugate vaccine, and 7149 persons (6128–10,724) with the 23-valent pneumococcal polysaccharide vaccines to prevent one invasive pneumococcal disease case [47]. All these values are much higher compared to those estimated for nirsevimab.

In summary, NNI for nirsevimab demonstrates a favorable comparison when evaluated alongside other childhood and adult vaccines that are part of the standard immunization schedule.

### 4.4. Use of Nirsevimab for RSV Prevention

Recommendations and strategies for universal prevention to protect all infants during their first RSV season with monoclonal antibodies have been implemented in several countries worldwide: since 2023/2024 in Spain, the USA, and France; then, since 2024/2025 in Chile, Australia, Canada, China, Austria, Belgium, Finland, Germany, Ireland, Luxembourg, the Netherlands, Portugal and Italy. Galicia, a region in northwest Spain, was among the first places globally to incorporate nirsevimab into its immunization schedule as a preventive measure against RSV. In this context, the NIRSE-GAL study provided real-world evidence on the effectiveness and public health impact of nirsevimab in preventing RSV-related LRTI hospitalizations, severe RSV-related LRTI, all-cause LRTI hospitalizations, and all-cause hospitalizations. Additionally, these findings enable the estimation of the NNI required to prevent one RSV-related LRTI hospitalization. The study provides policymakers and health authorities with strong evidence to inform the development of strategies for RSV prevention [19].

### 4.5. The Practical Implications of Using NNI in Health Policy Decision-Making

The NNI can significantly support health policy decision-making, particularly concerning the introduction and implementation of prophylactic measures, such as nirsevimab against RSV infection. Policymakers can explicitly utilize this NNI for quantifying programmatic effort and resource allocation. As a matter of fact, NNI directly quantifies the number of individuals who need to be immunized to prevent specific clinical outcomes, such as medical visits, emergency department visits, or hospitalizations caused by infectious diseases. For policymakers, this means they can clearly understand the scale of intervention required. Therefore, NNI numbers allow policymakers to estimate the required effort and plan for the necessary resources, including doses, personnel for administration, and logistics for distribution across regions. In addition, NNI serves as an easy and intuitive tool to demonstrate value and justify investment: this is crucial for gaining public acceptance and support for new health initiatives. Lastly, the favorable comparison of NNI for nirsevimab to NNV for many vaccines already included in national immunization programs provides a strong argument for its inclusion and continued funding.

Specifically, in Italy, while nirsevimab was not initially in the Italian NIP, its inclusion was recommended as universal immunoprophylaxis by the Board of the Calendar for Life and the Italian Society of Neonatology. Crucially, since the RSV season 2024/2025, all regions of Italy have implemented a preventive strategy with nirsevimab to protect all infants, supported by a specific fund from the Ministry of Health. The favorable NNI values, confirmed by studies, likely supported this policy decision.

### 4.6. Limitations of Study

One limitation of the methodology employed in this study is that it constitutes a narrative synthesis of the literature, rather than a formal systematic review. In addition, a risk-of-bias or study-quality assessment was not performed. Despite this approach, a comprehensive examination was conducted to identify the definition and application of the NNI within the context of infectious disease prevention. Although the methodology was a narrative synthesis, eight articles were identified that met the inclusion criteria. Of these, six studies utilized the NNI parameter to delineate the benefits of immunization and describe the advantages of monoclonal antibodies in RSV disease. This rigorous search suggests that, despite its narrative format, the study aimed to comprehensively identify and analyze all relevant studies describing the NNI in this specific context.

In addition, it was a challenge to directly compare the NNI values across different studies, as the sources indeed present a variety of denominators (e.g., age groups, per season) and a range of clinical outcomes (e.g., hospitalization, ICU admission, primary care visits). Some studies provided detailed methodologies (e.g., Finelli et al. used the reciprocal of absolute incidence rate reduction; Mallah et al. used Poisson regression or absolute risk reduction), while others did not. Therefore, performing a direct standardization (e.g., expressing all NNIs per 1000 person-seasons) or a simple meta-analysis is not possible. The review itself is a synthesis of existing literature rather than a re-analysis of raw data that would allow for such recalculations. Despite these variations and methodological differences, the overall conclusion across the sources is that the estimated NNI for nirsevimab is considered favorable when compared to the NNV for many other routine childhood and adult vaccines. This relative comparison is key to understanding its perceived benefit in public health contexts. In summary, while direct quantitative standardization or meta-analysis is beyond the scope of the provided review, a stratified narrative synthesis, as presented above, helps to clarify the specific outcomes being measured by NNI in different contexts.

## 5. Conclusions

The NNI can serve as a useful parameter to support public health decision-making: it can serve as an intuitive and easily understandable metric that can be used to communicate the value of an immunization intervention to various stakeholders, thereby supporting public health decision-making processes. However, it is essential to highlight a fundamental limitation of the metric: there is an absence of a shared threshold for interpreting results. There is not a universal, predefined number that intrinsically labels an NNI as acceptable or not, which can make it challenging to compare different prevention strategies. However, despite this limitation, the NNI is valuable for several reasons, and its acceptability can be inferred through contextual comparisons and its ability to quantify programmatic effort. As a matter of fact, the NNI is useful for quantifying the immunization effort required to prevent a single disease-related event, such as an RSV hospitalization. Programs that necessitate a lower NNI are generally considered more advantageous, as they imply more efficient use of resources to prevent an outcome. The NNI can be utilized as a parameter to demonstrate the cost-effectiveness ratio of a particular immunization program, which in turn can support the formulation of policy recommendations. Although an absolute threshold is lacking, the NNI for nirsevimab can be assessed by comparing it to the NNV for vaccines already included in national immunization programs. The studies indicate that the NNI for anti-RSV monoclonal antibodies is lower than the corresponding NNV for vaccines routinely included in childhood and adult immunization schedules, which is considered a favorable outcome. In essence, an NNI is deemed acceptable if the number of individuals to be immunized is relatively low and, crucially, if it compares favorably with established and accepted preventive interventions. This suggests that the programmatic effort required to prevent a single adverse event is efficient. However, it is fundamental that NNI calculations are applied with caution and interpreted critically. A significant limitation, similar to NNV, is that NNI does not account for the indirect effects of immunization, such as the reduction in secondary infections or the protective effects of herd immunity. Therefore, NNI might underestimate the overall public health benefits of an immunization program.

With the advent of novel monoclonal antibodies, new avenues for the prophylaxis of infectious diseases are emerging. The efficacy of these new monoclonal antibodies must be rigorously evaluated to determine their suitability for inclusion in national immunization programs, and NNI can serve as a key tool to achieve this when considered together with the uptake of the immunization program. Nirsevimab can be considered promising but still conditional on comprehensive economic and implementation assessments. Further studies are needed to determine the most appropriate use of this parameter and standardized methodologies for calculating and interpreting NNI, which are not yet commonly implemented.

However, at the current status, the estimated NNI of monoclonal antibodies against RSV is favorable and confirms their use in the first years of life for the prevention of RSV disease.

## Figures and Tables

**Table 1:** Definition of NNT, NNV, NNI.

	NNT (Number Needed to Treat)	NNV (Number Needed to Vaccinate)	NNI (Number Needed to Immunize)
Definition	The number of subjects needed to treat in order to prevent one outcome	The number of subjects needed to vaccinate in order to prevent one outcome	The number of subjects needed to be immunized to prevent one outcome
Population	Patients	Healthy subjects	Healthy subjects
Intervention	Medication	Vaccine	Vaccine or antibody
Outcomes	Recovery	Active prevention	Active or passive prevention

**Table 2:** The main characteristics of studies evaluating the NNI of monoclonal antibodies against RSV disease.

Authors	Journal	Year	Country	NNI Use	Context
Finelli et al. [35]	Vaccine	2020	USA	NNI to prevent one RSV-associated outpatient visit; NNI to prevent one RSV-associated lower respiratory tract infection (LRI) outpatient visit; RSV-associated hospitalizations	NNI to prevent RSV with extended half- life monoclonal antibody (EHL-mAb)
Francisco et al. [36]	Anales de Pediatrìa (English edition)	2023	Spain	NNI to prevent one RSV-associated hospitalization; NNI to prevent one RSV-associated hospitalization combined with severe disease	Francisco et al., in a Statement of the Spanish Society of Paediatric Infectious Disease (SEIP), provided recommendations for the administration of nirsevimab for prevention of RSV disease
O’Leary et al. [37]	Pediatrics	2023	USA	NNI with nirsevimab to prevent outpatient visits; NNI with nirsevimab to prevent emergency department visits; NNI with nirsevimab to prevent hospital admissions; NNI with nirsevimab to prevent Intensive Care Unit (ICU) admissions	The Advisory Committee on Immunization Practices (ACIP), a group of medical and public health experts that provides advice to the Centers for Disease Control and Prevention (CDC)
Mallah et al. [19]	The Lancet Infectious Diseases	2024	Spain	NNI to avoid one RSV-related lower respiratory tract infection (LRTI) hospitalization	Results of a population-based longitudinal study
Marcellusi et al. [39]	Global and Regional Health Technology Assessment	2025	Italy	NNI to prevent one RSV-related event per season	NNI is used to describe the potential benefits of a new prophylaxis strategy targeting all infants with nirsevimab compared to the current prophylaxis with palivizumab

**Table 3:** Summary table of the results of Finelli’s study [35].

Outcome	AGE	Immunization Efficacy	NNI
RSV-associated outpatient visit	0–5 months	50%	9–25 (95% CI 7–37)
		70%	7–18 (95% CI 5–25)
		90%	5–14 (95% CI 4–18)
	6–11 months	50%	8–22 (95% CI 6–33)
		70%	6–16 (95% CI 5–23)
		90%	5–12 (95% CI 4–17)
RSV-associated LRI outpatient visit	0–5 months	50%	24–44 (95% CI 17–76)
		70%	17–32 (95% CI 13–50)
		90%	14–25 (95% CI 10–37)
	6–11 months	50%	18–47 (95% CI 13–84)
		70%	13–33 (95% CI 10–56)
		90%	10–26 (95% CI 8–42)
RSV-associated hospitalizations	0–5 months	50%	52–118 (95% CI 39–138)
		70%	37–85 (95% CI 30–96)
		90%	29–66 (95% CI 24–74)
	6–11 months	50%	149–392 (95% CI 95–526)
		70%	107–280 (95% CI 73–360)
		90%	83–218 (95% CI 60–270)

**Table 4:** Strengths and limitations of NNI as a tool for assessment and decision process.

Strengths of NNI/NNV	Limits of NNI/NNV
NNI and NNV enable comparison to estimate the effort required to generate a significant public health impact, assuming high uptake and high coverage among the target population	NNI and NNV do not consider the indirect effects of vaccination
NNI and NNV are tools for estimating the cost associated with preventing a single disease event	Absence of defined threshold for interpretation of NNI and NNV
NNI can help to show the value of immunization to different stakeholders and decision makers	Possible underestimation for diseases with low R0
NNI enables comparison of different health strategies (not only preventive)	
